# Plasma Engineering of Co_4_N/CoN Heterostructure for Boosting Supercapacitor Performance

**DOI:** 10.3390/ma17143529

**Published:** 2024-07-16

**Authors:** Hong Li, Yunzhe Ma, Xulei Zhang, Xiuling Zhang, Lanbo Di

**Affiliations:** 1 College of Physical Science and Technology, Dalian University, Dalian 116622, China; lihong10@dlu.edu.cn (H.L.); myz19981028@163.com (Y.M.); xiulz@sina.com (X.Z.); 2 Sunstone Energy Co., Ltd., Jiayuguan 735100, China; xulei951105@163.com; 3 State Key Laboratory of Structural Analysis for Industrial Equipment, Dalian University of Technology, Dalian 116024, China; 4 Key Laboratory of Advanced Technology for Aerospace Vehicles of Liaoning Province, Dalian University of Technology, Dalian 116024, China

**Keywords:** supercapacitor electrode materials, metal–organic frameworks, transition metal nitrides, Co_4_N/CoN heterostructure

## Abstract

Supercapacitor electrode materials play a decisive role in charge storage and significantly affect the cost and capacitive performance of the final device. Engineering of the heterostructure of metal–organic framework (MOF)-derived transition metal nitrides (TMNs) can be conducive to excellent electrochemical performance owing to the synergistic effect, optimized charge transport/mass transfer properties, and high electrical conductivity. In this study, a Co_4_N/CoN heterostructure was incorporated into a nitrogen-doped support by radio-frequency (RF) plasma after simple pyrolysis of Co-based formate frameworks (Co-MFFs), with the framework structure well retained. Plasma engineering can effectively increase the ratio of Co_4_N in the Co_4_N/CoN heterostructure, accelerating the electron transfer rate and resulting in a rough surface due to the reduction effect of high-energy electrons and the etching effect of ions. Benefiting from the plasma modification, the obtained electrode material Co_4_N/CoN@C-P exhibits a high specific capacitance of 346.2 F·g^−1^ at a current density of 1 A·g^−1^, approximately 1.7 times that of CoN/Co_4_N@C prepared by pyrolysis. The specific capacitance of Co_4_N/CoN@C-P reaches 335.6 F·g^−1^ at 10 A·g^−1^, approximately 96.9% of that at 1 A·g^−1^, indicating remarkable rate capability. Additionally, the capacitance retention remains at 100% even after 1000 cycles, suggesting excellent cycling stability. The rational design and plasma engineering of the TMN heterostructures at the nanoscale are responsible for the excellent electrochemical performance of this novel composite material.

## 1. Introduction

With the rapid development of renewable and clean energy sources, energy storage systems play a significant role worldwide, particularly in the ever-increasing use of solar and wind energy systems [1,2,3,4]. Electrochemical supercapacitors (SCs) as energy storage devices have garnered considerable attention because of their rapid power delivery and recharging with high power density, stability, adaptability to harsh environments, and safety [5,6]. However, some challenges remain, such as their low energy density and high cost, severely limiting their further application [7,8]. Considering that the construction of electrode materials is a key aspect of supercapacitors for enhancing their performance, developing advanced composite materials with cost efficiency using multiple technologies is highly desirable [9,10,11]. 

Metal–organic frameworks (MOFs) are a class of novel crystalline porous materials with controllable compositions, diverse structures, and high specific surface areas. MOFs can be converted into porous carbon materials through a simple pyrolysis treatment, making them widely applicable in energy storage applications [12,13]. MOF-derived porous carbon materials have a porous structure and large specific surface area. Simultaneously, when using Zn-, Fe-, and Co-based MOFs as electrodes for supercapacitors, the presence of metal centers may enhance their ability to store energy through pseudocapacitance [14,15,16]. These remarkable structural advantages enable the great potential of MOF-derived carbon as high-performance energy materials, which, to date, have been applied in the field of energy storage systems. However, their relatively low specific capacitances limit their practical applications [17,18,19]. In recent years, considerable effort has focused on transition metal nitrides (TMNs) (especially Co_4_N) and their diverse compositions owing to their excellent electrical conductivity, chemical corrosion resistance, chemical reactivity, and electrochemical stability [20,21,22]. Typically, TMNs contain metallic, ionic, and covalent bonds. Metal–nitride bonding expands the parent metal lattice and narrows its d-bands [5,23,24,25]. Owing to the scarcity of the d-band and increased density of states (DOS) near the Fermi level of TMNs, they display characteristics similar to those of noble metals in electrocatalysis, which enables them to effectively attract reactants/ions to their surfaces, thus making them appealing options for electrochemical energy storage and conversion [26,27]. Therefore, maximizing the overall electrochemical performance by carefully integrating the advantages of the Co_x_N and MOF structures by exploiting the intrinsic characteristics of different materials is beneficial.

Recently, significant progress has been made in heterostructure engineering, a highly effective approach for achieving optimal control over electrochemical activity and mass and charge transport. The advantages of heterostructures can be primarily attributed to synergistic effects, strain effects, and electronic interactions [28]. The synergistic effect, one of the most important characteristics of heterostructures, is induced by heterointerfaces and multiple components. In general, the interfacial bonding in these heterostructures can effectively facilitate electron transfer and enhance electrical conductivity. In addition to conductivity, other qualities, such as hydrophilicity, the density of active sites, electrochemical stability, mechanical properties, and mass-transfer efficiency, can be adjusted [29]. Moreover, owing to their diverse chemical compositions and crystal structures, the arrangement of atoms at the heterointerface differs from that in bulk materials, causing lattice strain, including both tensile and compressive strains. Typically, heterostructures subjected to tensile strain cause a decrease in the overlap of the d-orbitals, reducing the bandwidth, which, in turn, causes an increase in the d-band center and enhances the adsorption energy [30]. Finally, heterostructured materials inevitably alter the electronic structure in each phase along their interfaces—beneficial for electronic interactions and surface electron modulation [31,32]. Therefore, modifying heterostructures through chemical/physical methods can further optimize the electronic structure and charge transport/mass transfer properties in the electrochemical process and enhance the capacitive performance. 

Cold plasma is a rapid, facile, and environmentally friendly method for treating different materials, and it has consequently garnered growing research interest [33,34,35]. Among the diverse plasmas, radio-frequency (RF) plasma can generate abundant high-energy electrons, ions, and active species at low pressures with a low gas temperature and precisely induce nanoscale reactions in electrode materials, achieving reduction, doping, etching, and exfoliation [33]. For a typical argon ICP discharge, the high-energy electrons present in the plasma can realize the reduction of cobalt ions to a lower valence, while the ions with high energy can etch the material surface by physical bombardment, making the surface structure rougher, exposing more Co_x_N active sites, and promoting the formation of heterostructures [36,37]. Additionally, a low gas temperature is beneficial for preventing structural damage to the MOFs and particle agglomeration.

Based on the above understanding, this study utilizes a plasma modification strategy to regulate the contents of Co_4_N/CoN heterostructures and the surface morphology of MOF materials, resulting in a Co_4_N/CoN@C-P material. This material combines the advantages of porous MOFs and N-doped carbon structures, with the Co_4_N/CoN heterostructure accelerating the charge transfer between the crystal interface, enhancing the electrical conductivity. Thus, the obtained Co_4_N/CoN@C-P exhibits excellent electrochemical performance in supercapacitors. 

## 2. Experimental Section

### 2.1. Preparation of the Electrode Materials

#### 2.1.1. Preparation of Co-MFF

Co-MFFs were prepared using the liquid-phase precipitation method. The basic process was as follows: First, 0.58 g of cobalt nitrate and 0.5 g of polyethylene glycol (PEG) were weighed and dissolved in 10 mL methanol; the dissolved solution is denoted as solution A. Then, 1.01 g of ammonium formate and 0.5 g of PEG were weighed and dissolved in 10 mL methanol to obtain solution B; solution B was stirred at a constant speed. After all solids in solution B were dissolved, solution A was slowly added dropwise to solution B. After continuous magnetic stirring at room temperature for 1 h, a pink solid powder was separated after 24 h, centrifuged with ethanol five times, and dried in a vacuum drying oven at 55 °C for 7 h to obtain the Co-MFF sample [38].

#### 2.1.2. Preparation of CoN/Co_4_N@C

A Co-MFF sample (2 g) was pyrolyzed in a tube furnace. An ammonia/argon gas mixture (*V*_NH3:_*V*_Ar_ = 1:9) was utilized, with a total flow rate of 50 mL·min^−1^, pyrolysis temperature of 450 °C, heating rate of 5 °C·min^−1^, and pyrolysis time of 2 h. After pyrolysis and natural cooling to room temperature, the sample was collected, weighed, and labeled as CoN/Co_4_N@C. 

#### 2.1.3. Preparation of Co_4_N/CoN@C-P 

A CoN/Co_4_N@C sample (400 mg) was treated in a low-pressure radio frequency (RF)-based inductively coupled plasma (ICP) setup. After the chamber vacuum was pumped to 0.1 Pa, argon (or nitrogen) was fed into the chamber for 20 min, the RF automatic impedance matcher and RF power supply were turned on, the CoN/Co_4_N@C was processed at a gas pressure range of 20–60 Pa under RF power of 200–350 W for 2–8 min, and then the RF power was turned off. When the quartz tube decreased to room temperature, the sample was collected, weighed, and labeled as Co_4_N/CoN@C-P. It should be noted that the discharge conditions mentioned later in this work refer to argon plasma with 40 Pa, 300 W, and 6 min unless otherwise specified.

### 2.2. Electrochemical Measurement

Cyclic voltammetry (CV), galvanostatic charge–discharge (GCD), and electrochemical impedance spectroscopy (EIS) were performed using an electrochemical workstation (CHI760E, CH Instruments, China). In the tests, the CV scanning rate was 10–50 mV·s^−1^ and the GCD current density was 1–10 A·g^−1^. The EIS tests were performed at a frequency of 0.01–10^5^ Hz with 5 mV AC perturbation. According to the GCD curve, the specific capacitance of the sample can be calculated as

(1) $$C=\frac{I∆t}{m∆V}$$

where *I* is the current, Δ*t* is the discharge time, *m* is the mass of the active substance, and Δ*V* is the potential difference. 

The coulombic efficiency can be calculated as

(2) $$\eta =\frac{{\Delta t}_{d}}{{\Delta t}_{c}}$$

where *η* is the coulombic efficiency, and Δ*t*_d_ and Δ*t*_c_ are the discharge and charging time, respectively.

### 2.3. Characterization

The species composition and phase structure of the samples were analyzed by X-ray diffraction (XRD) (DX-2700, Dandong, China), and the diffraction source was a Cu-Kα ray operated at a voltage of 40 kV and a current of 30 mA. The morphologies of the samples were investigated using scanning electron microscopy (SEM) (ZEISS Gemini 300, Jena, Germany) at an accelerating voltage of 5 kV. High-resolution transmission electron microscopy (HRTEM) (Talos Model F200X, Waltham, MA, USA) was used to observe the morphologies of the samples at an accelerating voltage of 120 kV. The surface chemistry valence states of the composites were analyzed by XPS (Thermo Scientific Model K-Alpha, Waltham, MA, USA). The excitation source was an X-ray source with a monochromatic Al target (photon energy of 1486.6 eV, 150 W), with C1s (binding energy = 284.8 eV) used to correct the displacement of each element in the test during the analysis.

## 3. Results and Discussion

### 3.1. Electrochemical Performance

**Figure 1:**
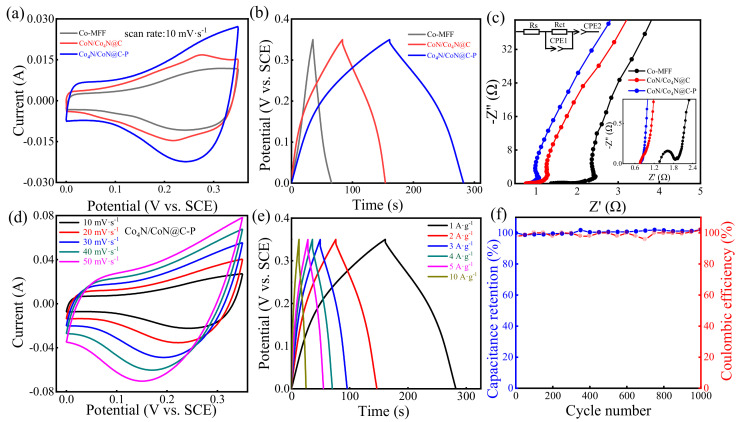
Electrochemical performance of Co-MFF, CoN/Co_4_N@C, and Co_4_N/CoN@C-P electrodes in 6 M KOH electrolyte with a three-electrode system. (**a**) CV curves at 10 mV·s^−1^; (**b**) GCD curves at 1 A·g^−1^; (**c**) EIS curves; (**d**) CV profiles of Co_4_N/CoN@C-P at different scan rates; (**e**) GCD curves of Co_4_N/CoN@C-P at different current densities; (**f**) Capacitance retention and coulombic efficiency of the Co_4_N/CoN@C-P over 1000 cycles at 1 A·g^−1^.

At 300 W and 40 Pa, the Co_4_N/CoN@C-P samples treated by argon RF ICP for 6 min exhibited the best performance (Figures S2–S4). Co-MFF, CoN/Co_4_N@C, and Co_4_N/CoN@C-P were fabricated into electrode sheets, and their electrochemical performance was measured in a 6 M KOH electrolyte using a three-electrode system. Figure 1a shows the CV curves of Co-MFF, CoN/Co_4_N@C, and Co_4_N/CoN@C-P at a scan rate of 10 mV·s^−1^ and a voltage range of 0–0.35 V. Co_4_N/CoN@C-P exhibited a higher capacitive current response and a larger CV integration area, implying a larger charge storage capacity. Figure 1b shows the GCD curves of the three electrode materials at a current density of 1 A·g^−1^ and a voltage range of 0–0.35 V. Compared to the Co-MFF and CoN/Co_4_N@C, Co_4_N/CoN@C-P had longer charge/discharge times, consistent with the trend of the CV curves. Moreover, the specific capacitance of Co_4_N/CoN@C-P was 346.2 F·g^−1^, much higher than that of CoN/Co_4_N@C (208.5 F·g^−1^) under the same conditions. Figure 1c shows the EIS results for the three electrode materials, with the upper inset showing the equivalent circuit diagram and the lower inset showing the local enlarged image. The fitted Nyquist plots of Co-MFF, CoN/Co_4_N@C, and Co_4_N/CoN@C-P are composed of a semicircular arc and a straight line. The semicircular arc observed in the high-frequency region represents the redox reaction occurring at the interface between the electrode and the electrolyte. The straight line observed in the low-frequency region indicates the diffusion of ions in the electrode. An equivalent circuit was established to better interpret the Nyquist plots (upper inset in Figure 1c), and all the fitted results are summarized in Table S1. The intersection of the semicircular arc in the high-frequency region and the real axis represents the series resistance (*R*_s_), and the diameter of the semicircular arc represents the charge transfer resistance (*R*_ct_). Constant phase element 1 connected in parallel with *R*_ct_ represents a Faraday capacitor (*C*_F_), and constant phase element 2 connected in series represents an electric double-layer capacitor (*C*_dl_). As shown in Table S1, *R*_s_ and *R*_ct_ of Co_4_N/CoN@C-P are 0.73 and 0.28 Ω, respectively, which are lower than those of CoN/Co_4_N@C (*R*_s_ = 0.73 Ω, *R*_ct_ = 0.34 Ω) and Co-MFF (*R*_s_ = 1.38 Ω, *R*_ct_ = 0.54 Ω). In short, the Co_4_N/CoN@C-P material has the largest slope in the low-frequency region and a smaller arc radius in the high-frequency region, indicating that the sample has minimal resistance, which, in turn, indicates that the electrode material has good electrical conductivity—conducive to rapid charge transfer. Figure 1d exhibits the CV curves of Co_4_N/CoN@C-P at 10–50 mV·s^−1^ scan rates. Although the absence of narrow redox peaks is observed, the CV curve shape is not an ideal rectangle, indicating a charge contribution from a pseudocapacitance mechanism [39]. By increasing the scan rate, the redox peaks gradually separate, while the shape of the CV curve remains unchanged, implying faradaic pseudocapacitance behavior, excellent reversibility, and faster charge transfer [39,40,41]. Generally, capacitance decreases with increasing current density owing to the diffusion effect. However, as shown in the GCD plot of Co_4_N/CoN@C-P given in Figure 1e at 1–10 A·g^−1^, the specific capacitance of Co_4_N/CoN@C-P at 10 A·g^−1^ is approximately 96.9% of that at a current density of 1 A·g^−1^, indicating excellent energy storage performance and rate capability. The cyclic stability of Co_4_N/CoN@C-P is also evaluated in the voltage range of 0–0.35 V and a current density of 1 A·g^−1^, as shown in Figure 1f. After 1000 cycles, the capacitance retention reached 100%, and the coulombic efficiency was consistently maintained at nearly 100%, indicating excellent cycling stability and a favorably reversible charge storage and delivery process. The improved capacitive performance of Co_4_N/CoN@C-P can be attributed to its unique morphology and structure.

### 3.2. Morphological and Structural Characterizations

**Figure 2:**
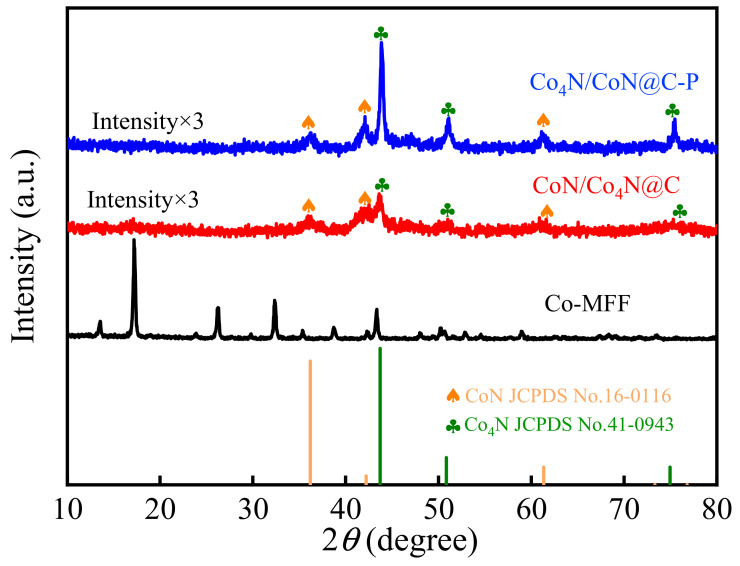
XRD patterns of Co-MFF, CoN/Co_4_N@C, and Co_4_N/CoN@C-P.

XRD characterization tests were conducted for Co-MFF, CoN/Co_4_N@C, and Co_4_N/CoN@C-P, as shown in Figure 2, to investigate the crystal phase structures of the different electrode materials. The positions of the Co-MFF diffraction peaks at 2*θ* = 13.97°, 17.69°, 26.66°, 32.81°, 35.81°, 39.14°, 42.77°, 43.76°, and 48.47° are consistent with those of Co-MFF in the literature [42,43]. The diffraction peaks of CoN/Co_4_N@C at 2*θ* = 44.59°, 51.70°, and 76.10° correspond to the characteristic diffraction peaks of Co_4_N at the (111), (200), and (220) crystal faces (JCPDS: 41-0943) of the cubic phase, respectively. The diffraction peaks at 2*θ* = 36.19°, 42.19°, and 61.34° correspond to the characteristic diffraction peaks of the (111), (200), and (220) crystal faces (JCPDs: 16-0116) of CoN. The characteristic diffraction peaks of Co_4_N and CoN in the cubic phase are also observed in Co_4_N/CoN@C-P. Compared to CoN/Co_4_N@C, the characteristic peak of Co_4_N in Co_4_N/CoN@C-P is higher and sharper than that of CoN, indicating that the ratio of Co_4_N in Co_4_N/CoN@C-P increased after the argon RF plasma treatment. This maybe because the high-energy electrons generated in the plasma have strong reduction ability, and the high-valence state of cobalt ions can be reduced to a lower one. 

**Figure 3:**
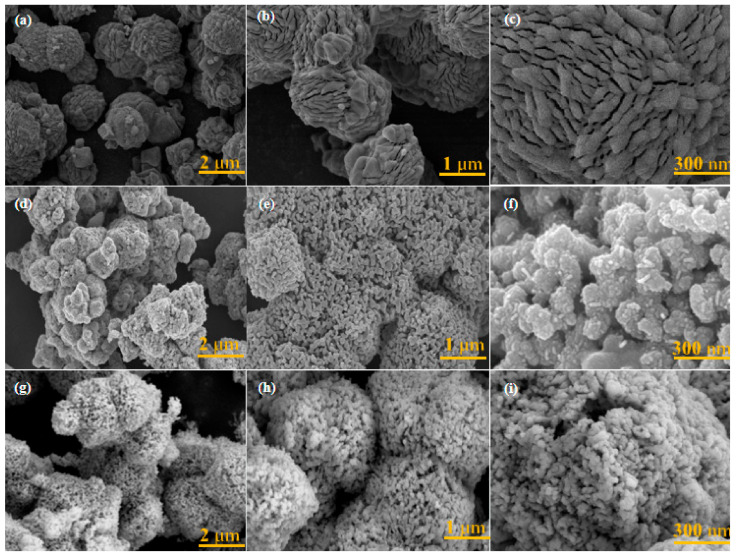
Typical SEM images of (**a**–**c**) Co-MFF, (**d**–**f**) CoN/Co_4_N@C, and (**g**–**i**) Co_4_N/CoN@C-P.

SEM tests were conducted to further examine the surface morphologies and microstructures of the Co-MFF, CoN/Co_4_N@C, and Co_4_N/CoN@C-P electrode materials. Figure 3a–c show the surface morphologies of the Co-MFF at different scales. The sample presents an octahedral morphology, signifying the successful synthesis of Co-MFF. Figure 3d–f show the surface morphologies of CoN/Co_4_N@C at different scales. The existence of the octahedral morphology and a porous structure indicates that the framework of Co-MFF was stable after ammonia/argon pyrolysis. The structural confinement effect of the MFF framework helps stabilize the CoN and Co_4_N nanoparticles and avoid nanoparticle agglomeration. Figure 3g–i show the surface morphology of Co_4_N/CoN@C-P, maintaining the basic morphology of the MFF, with the frame structure still in good condition; however, the surface of the treated sample became rougher because the high-energy argon ions generated in the plasma can strongly physically bombard the surface of the material, causing the surface of the material to be etched. A rough surface topography is usually conducive to full contact between the electrode surface and the electrolyte, facilitating ion mass transfer, reducing the electrolyte diffusion distance, and further improving the capacitance of the electrode material [44].

**Figure 4:**
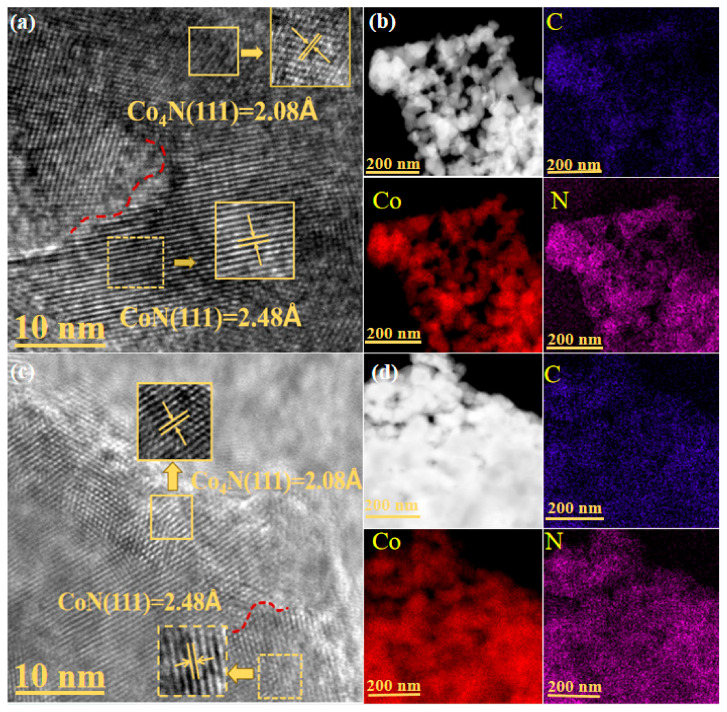
HRTEM images and EDS elemental mappings of the Co, N, and C elements for (**a**,**b**) CoN/Co_4_N@C and (**c**,**d**) Co_4_N/CoN@C-P (The red dash lines indicate the heterointerface).

Clear lattice fringes of 2.48 A and 2.08 A, corresponding to the CoN (111) and Co_4_N (111) crystal faces, were observed in the HRTEM images of the CoN/Co_4_N@C and Co_4_N/CoN@C-P (Figure 4a,c), respectively, and exhibited an apparent heterointerface, indicating the formation of the Co_4_N and CoN heterostructures in the CoN/Co_4_N@C and Co_4_N/CoN@C-P. Relevant research results show that the redistribution of electrons at the heterointerface can promote a synergistic effect between Co_x_N heterostructures, resulting in a higher electrical conductivity of the heterostructures—conducive to enhancing the electrochemical properties of the prepared electrode materials [45,46,47]. Indeed, as shown in Figures S1 and S2, the Co_4_N@C-P with a single component of Co_4_N obtained by treating CoN/Co_4_N@C with nitrogen plasma exhibited lower electrochemical performance. In addition, as shown in Figure 4b,d, the energy-dispersive X-ray spectroscopy (EDS) elemental mapping images demonstrate that Co, N, and C were uniformly distributed in the CoN/Co_4_N@C and Co_4_N/CoN@C-P. 

**Figure 5:**
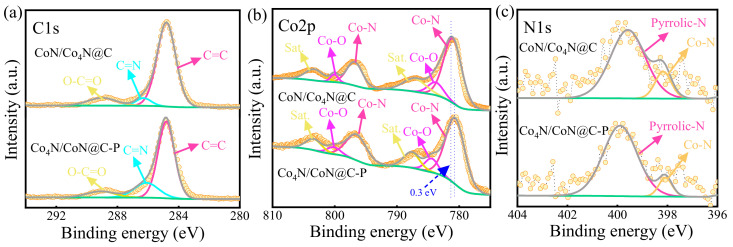
XPS spectra of (**a**) C1s, (**b**) Co2p, and (**c**) N1s for CoN/Co_4_N@C and Co_4_N/CoN@C-P.

Considering that the plasma-induced surface modification of materials usually affects the electron valence states of surface atoms, the chemical valence states and surface element compositions of the CoN/Co_4_N@C and Co_4_N/CoN@C-P were examined by XPS. Figure 5 shows the XPS energy spectra of C1s, Co2p, and N1s of the CoN/Co_4_N@C and Co_4_N/CoN@C-P materials (with C1s 284.8 eV as the standard peak). In Figure 5a (C1s spectra), the peaks at the binding energies of 284.8, 286.3, and 289.1 eV correspond to the convolution peaks of the C=C, C-N, and N-C=O bonds, respectively. The CoN/Co_4_N@C and Co_4_N/CoN@C-P had some N doped into the carbon layer structure. However, the increase in the C-N bond peak area in the Co_4_N/CoN@C-P compared to that of the CoN/Co_4_N@C indicates an increase in the proportion of nitrogen doping. This enhanced nitrogen doping helped improve the polarity of the electrode material, ensuring full contact with the electrolyte, and effectively improving the specific capacitance and conductivity of the electrode material.

Figure 5b shows the Co2p spectra of the CoN/Co_4_N@C and Co_4_N/CoN@C-P. The CoN/Co_4_N@C signals at 781.1 and 796.8 eV are attributed to the Co-N bond in the CoN compound, while the signals at 787.2 and 803.3 eV are attributed to the Co2p satellite peak. The signals of the Co_4_N/CoN@C-P sample at 780.8 and 796.5 eV are attributed to the Co-N bond in the CoN compound, with satellite peaks at 787.6 and 803.6 eV [39]. The two spin-splitting peaks at 784.2 and 801.1 eV may be related to sample surface oxidation [48,49]. Based on a semi-quantitative analysis of the samples, the ratios of Co/C in the CoN/Co_4_N@C and Co_4_N/CoN@C-P are approximately 1:7.85 and 1:7.68, respectively, indicating that the ratios of Co/C in the samples treated by plasma modification are relatively stable. Combined with the XRD results, the valence state of Co and the relative contents of CoN and Co_4_N changed under the effect of plasma reduction. Moreover, compared to the binding energy of the Co-N bond in the CoN/Co_4_N@C, the value of the Co_4_N/CoN@C-P shifted downward by 0.3 eV, indicating a strong electronic interaction between Co_4_N and CoN, eventually resulting in charge redistribution between the heterogeneous interfaces—conducive to the improvement of capacitive performance [50,51]. 

Figure 5c shows the energy spectra of N1s. The two binding energy peaks of 398.2 and 399.7 eV in the CoN/Co_4_N@C are attributed to the Co-N and pyrrole nitrogen peaks, respectively, further confirming the formation of metal nitrides. The binding energy peaks of 398.1 and 399.9 eV in the Co_4_N/CoN@C-P correspond to the Co-N and pyrrole nitrogen peaks, respectively. These N dopants help enhance the conductivity of the electrode, promote electron transport performance, and contribute to the additional pseudocapacitance [52,53,54].

The specific surface area (*S*_BET_) of the samples plays a key role in the charge transfer and storage. The BET of the samples was also obtained, as illustrated in Table S2. The *S*_BET_ of the Co-MFF was 65.0 m^2^·g^−1^ in this work. The *S*_BET_ values of the CoN/Co_4_N@C, obtained by the pyrolysis of Co-MFF, and the Co_4_N/CoN@C-P, prepared by the plasma processing of CoN/Co_4_N@C, were only 17.7 and 7.0 m^2^·g^−1^. The *S*_BET_ value of Co_4_N/CoN@C-P was smaller than that of CoN/Co_4_N@C. Therefore, it further verifies that the Co_4_N/CoN heterostructure instead of the *S*_BET_ plays a vital role in boosting supercapacitor performance.

### 3.3. Discussion

In summary, a rapid, simple, and mild plasma surface modification method was employed to treat Co-MFF after simple pyrolysis. This process enables nitrogen doping of the support when constructing and regulating the Co_4_N/CoN heterostructure. From the electrochemical performance results of the three materials (Figure 1), the Co_4_N/CoN@C-P electrode material prepared by plasma-assisted pyrolysis exhibited superior electrochemical performance compared to the electrode materials before and after the pyrolysis of Co-MFF, exhibiting a specific capacitance of 346.2 F·g^−1^ at a current density of 1 A·g^−1^, approximately 1.7 times that of CoN/Co_4_N@C prepared by pyrolysis. Furthermore, Co_4_N/CoN@C-P demonstrated a remarkable rate capability and excellent cycling stability. The improved capacitive performance of Co_4_N/CoN@C-P can be attributed to its optimized heterostructure and the rough surface of the modified MFF structure.

The SEM results indicate that the framework structure of the MFF remained unchanged after RF plasma treatment—beneficial for the dispersion of cobalt–nitrogen nanoparticles. The HRTEM images show the formation of Co_4_N and CoN heterostructures in both CoN/Co_4_N@C and Co_4_N/CoN@C-P. The XRD results reveal that the relative contents of Co_4_N and CoN changed significantly after plasma treatment owing to the strong reduction ability of the high-energy electrons generated in the plasma, which generated more Co_4_N through its reduction effect. The synergistic effect between the increased Co_4_N and CoN heterostructures facilitated a rapid charge transfer during the charge/discharge process, resulting in a higher electrical conductivity of the heterostructures and significantly enhancing the electrochemical performance.

Additionally, the SEM results indicate that the material surface became rougher after the plasma treatment, owing to the etching effect caused by the high-energy argon ions bombarding the material surface in the plasma, which can expose more Co_x_N active sites. Furthermore, the porous nature of the MFF material facilitates electron transport, and the etching effect of the plasma promotes the formation of a heterostructure [36]. The XPS results show that plasma surface modification can increase nitrogen doping into the carbon in Co_4_N/CoN@C-P, which promotes better contact between the electrode and electrolyte and improves the conductivity of the material, thus enhancing the electrochemical performance of the Co_4_N/CoN@C-P electrode material. Finally, the BET results further verify that the Co_4_N/CoN heterostructure instead of the *S*_BET_ plays a vital role in boosting supercapacitor performance.

## 4. Conclusions

A Co_4_N/CoN@C-P electrode material with high electrochemical performance was prepared by an integrated strategy combining plasma modification and the simple pyrolysis of Co-MFF. This approach successfully promoted the nitrogen doping of the carbon support after plasma treatment, formed a Co_4_N/CoN heterostructure, and increased the proportion of Co_4_N. The electrochemical performance of the electrode materials was measured using a three-electrode system, which revealed that the specific capacitance of Co_4_N/CoN@C-P reached up to 346.2 F·g^−1^, approximately 1.7 times that of the CoN/Co_4_N@C prepared by pyrolysis only. The characterization results indicate that the framework structure of the Co-MFF was well retained after plasma modification—beneficial for the dispersion of cobalt nitride nanoparticles. The porous nature of the MFF material also facilitated charge transfer. Both the CoN/Co_4_N@C and Co_4_N/CoN@C-P electrode materials contained Co_4_N and CoN heterostructures; however, the plasma treatment significantly increased the Co_4_N content in the heterostructure, which was due to the strong reduction ability of the high-energy electrons generated in the plasma. Moreover, bombardment by high-energy Ar ions rendered the material surface rougher, exposing more Co_x_N active sites—beneficial for rapid charge transfer. Finally, more nitrogen atoms were doped into the carbon in Co_4_N/CoN@C-P, enhancing the contact between the electrode material and the electrolyte, improving the conductivity of the carbon and thus enhancing the electrochemical charge transfer, further improving the electrochemical performance of the Co_4_N/CoN@C-P material. This research demonstrates that plasma engineering of metal–nitride heterostructures derived from MOFs can effectively enhance electrochemical performance, thus providing valuable insights into the controllable preparation of other high-performance electrochemical materials. 

## Data Availability

The original contributions presented in the study are included in the article/Supplementary Materials, further inquiries can be directed to the corresponding author.

## Supplementary Materials

The following supporting information can be downloaded at: https://www.mdpi.com/article/10.3390/ma17143529/s1. Figure S1: XRD patterns of Co_4_N/CoN@C-P (Ar) and Co_4_N@C-P (N_2_) prepared at 40 Pa, 300 W and discharge time of 6 min; Figure S2: Electrochemical performance for different discharge atmosphere at a fixed power of 300 W and discharge time of 6 min, with different gas pressures of 20, 40, and 60 Pa. (a) CV curves at 10 mV·s^-1^, (b) GCD curves at 1 A·g^-1^, (c) EIS curves of Co_4_N@C-P(N_2_); (d) CV curves at 10 mV·s^-1^, (e) GCD curves at 1 A·g^-1^, (f) EIS curves of Co_4_N/CoN@C-P(Ar); Figure S3: Electrochemical performance of Co_4_N/CoN@C-P(Ar) prepared at 300 W and 40 Pa, with different discharge time of 2, 4, 6, and 8 min. (a) CV curves at 10 mV·s^-1^; (b) GCD curves at 1 A·g^-1^; (c) EIS curves; Figure S4: Electrochemical performance of Co_4_N/CoN@C-P(Ar) prepared at 40 Pa and discharge time of 6 min, with different discharge powers of 200, 250, 300, and 350 W. (a) CV curves at 10 mV·s^-1^; (b) GCD curves at 1 A·g^−1^; (c) EIS curves. Table S1: Equivalent circuit parameters obtained from Nyquist plots of Co-MFF, CoN/Co_4_N@C and Co_4_N/CoN@C-P; Table S2: The specific surface areas (*S*_BET_), pore volumes (*V*_p_), and pore diameters (*D*_p_) of Co-MFF, CoN/Co_4_N@C, and Co_4_N/CoN@C-P.
