# Supplementary material for: Plasma Engineering of Co4N/CoN Heterostructure for Boosting Supercapacitor Performance
Source: Materials (Basel). 2024 Jul 16;17(14):3529. doi: 10.3390/ma17143529 (PMC11278462; doi:10.3390/ma17143529)
Supplement: Supplementary file 1 [file materials-17-03529-s001.zip › materials-3084297-supplementary.pdf]

Supporting Information

Plasma engineering of Co_4N/CoN heterostructure for boosting supercapacitor performance

Hong Li [1], Yunzhe Ma [1], Xulei Zhang [2], Xiuling Zhang [1] and Lanbo Di [1,3,4,*]

1 College of Physical Science and Technology, Dalian University, Dalian 116622, China

2 Sunstone Energy Co., Ltd., Gansu 735100, China

3 State Key Laboratory of Structural Analysis for Industrial Equipment, Dalian University of Technology, Dalian 116024, China

4 Key Laboratory of Advanced Technology for Aerospace Vehicles of Liaoning Province, Dalian University of Technology, Dalian 116024, China

* Correspondence: dilanbo@163.com (L. B. Di).

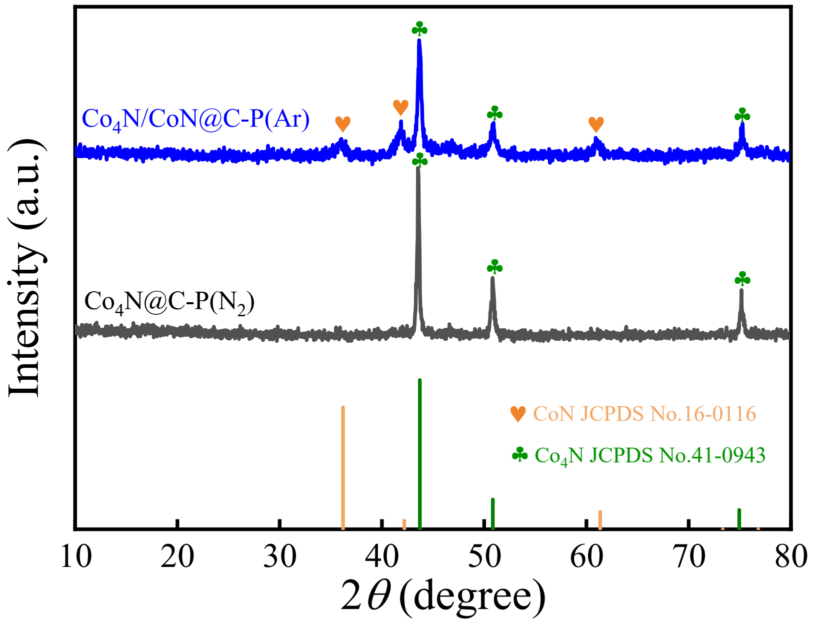

Figure S1. XRD patterns of Co_4N/CoN@C-P (Ar) and Co_4N@C-P (N_2) prepared at 40 Pa, 300 W and discharge time of 6 min.

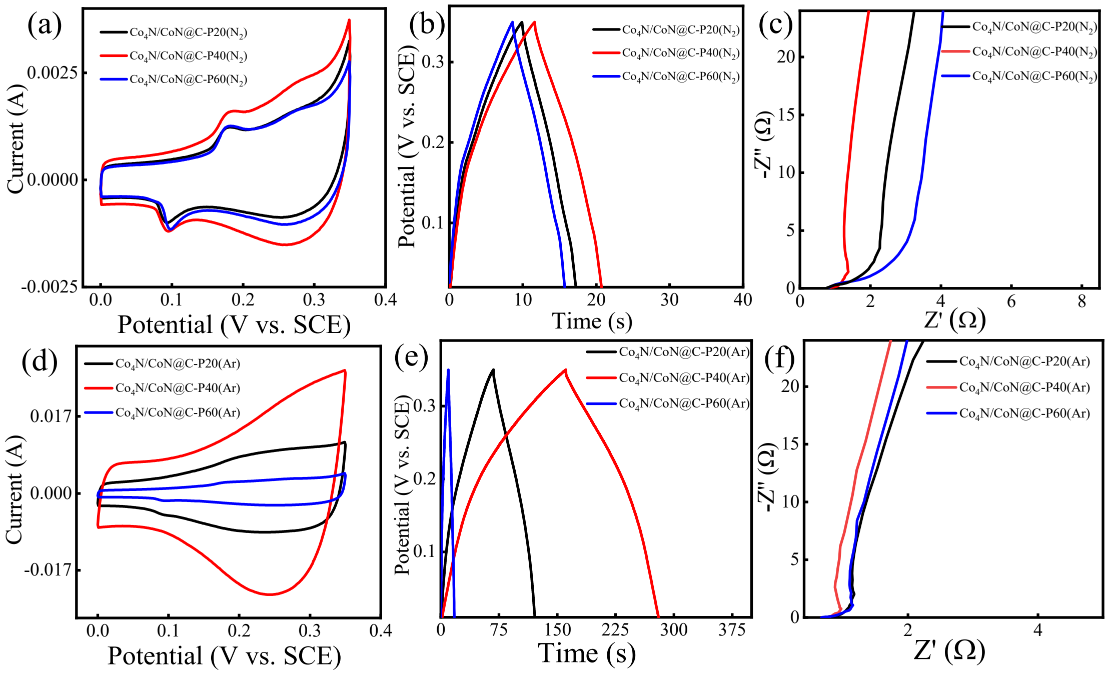

Figure S2. Electrochemical performance for different discharge atmosphere at a fixed power of 300 W and discharge time of 6 min, with different gas pressures of 20, 40, and 60 Pa. (a) CV curves at 10 mV·s^{-1}, (b) GCD curves at 1 A·g^{-1}, (c) EIS curves of Co_4N@C-P(N_2); (d) CV curves at 10 mV·s^{-1}, (e) GCD curves at 1 A·g^{-1}, (f) EIS curves of Co_4N/CoN@C-P(Ar).

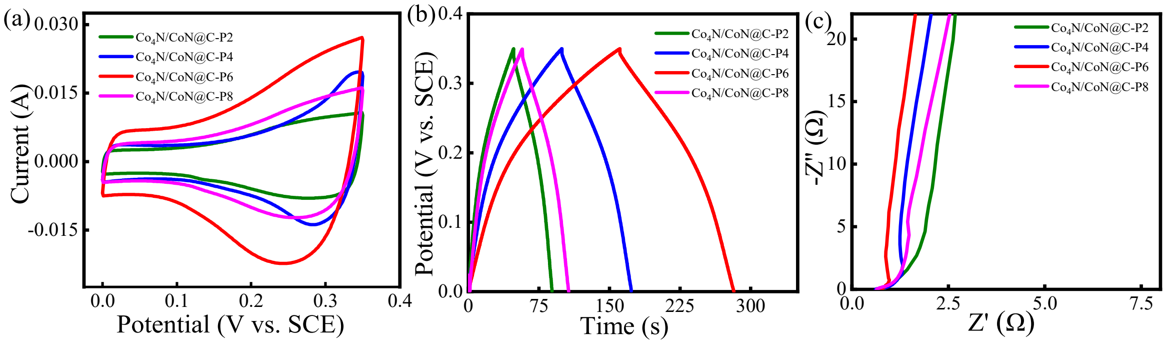

Figure S3. Electrochemical performance of $Co_4N/CoN@C$-P(Ar) prepared at 300 W and 40 Pa, with different discharge time of 2, 4, 6, and 8 min. (a) CV curves at 10 mV·s^{-1}; (b) GCD curves at 1 A·g^{-1}; (c) EIS curves.

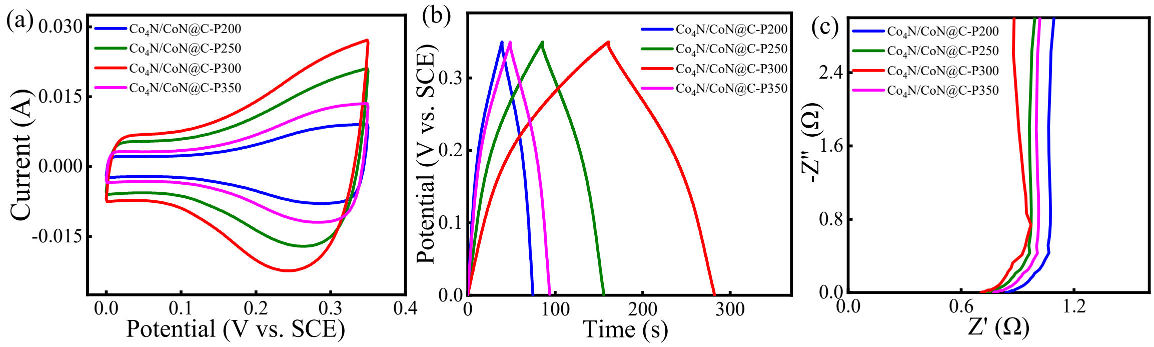

Figure S4. Electrochemical performance of $Co_4N/CoN@C$-P(Ar) prepared at 40 Pa and discharge time of 6 min, with different discharge powers of 200, 250, 300, and 350 W. (a) CV curves at 10 mV·s^{-1}; (b) GCD curves at 1 A·g^{-1}; (c) EIS curves.

Table S1. Equivalent circuit parameters obtained from Nyquist plots of Co-MFF, $CoN/Co_4N@C$ and $Co_4N/CoN@C$-P.

	R_s(Ω)	R_{ct}(Ω)	CPE1	CPE2
Co-MFF	1.38	0.54	0.0434	0.1583
Error (%)	3.59	5.94	6.0140	0.8009
$CoN/Co_4N@C$	0.73	0.34	0.0451	0.0416
Error (%)	1.48	5.39	3.5010	0.5594
$Co_4N/CoN@C$-P	0.73	0.28	0.0510	0.0690
Error (%)	1.29	4.26	7.2620	0.7953

Table S2. The specific surface areas (S_{BET}), pore volumes (V_p), and pore diameters (D_p) of Co-MFF, CoN/Co_4N@C, and Co_4N/CoN@C-P.

	S_{BET} ($m^2 \cdot g^{-1}$)	V_p ($cm^{-3} \cdot g^{-1}$)	D_p (nm)
Co-MFF	65.0	0.1905	3.777
CoN/Co_4N@C	17.7	0.0405	3.382
Co_4N/CoN@C-P	7.0	0.0740	42.661
